# Overexpression of the ATPase Inhibitory Factor 1 Favors a Non-metastatic Phenotype in Breast Cancer

**DOI:** 10.3389/fonc.2017.00069

**Published:** 2017-04-10

**Authors:** Lucía García-Ledo, Cristina Nuevo-Tapioles, Carmen Cuevas-Martín, Inmaculada Martínez-Reyes, Beatriz Soldevilla, Lucía González-Llorente, José M. Cuezva

**Affiliations:** ^1^Departamento de Biología Molecular, Centro de Biología Molecular Severo Ochoa (CSIC-UAM), Centro de Investigación Biomédica en Red de Enfermedades Raras CIBERER-ISCIII, Instituto de Investigación Hospital 12 de Octubre, Universidad Autónoma de Madrid, Madrid, Spain

**Keywords:** mitochondria, ATP synthase, ATPase inhibitory factor 1, gene expression analysis, breast cancer, extracellular matrix, cellular migration, cellular invasion

## Abstract

Partial suppression of mitochondrial oxidative phosphorylation and the concurrent activation of aerobic glycolysis is a hallmark of proliferating cancer cells. Overexpression of the ATPase inhibitory factor 1 (IF1), an *in vivo* inhibitor of the mitochondrial ATP synthase, is observed in most prevalent human carcinomas favoring metabolic rewiring to an enhanced glycolysis and cancer progression. Consistently, a high expression of IF1 in hepatocarcinomas and in carcinomas of the lung, bladder, and stomach and in gliomas is a biomarker of bad patient prognosis. In contrast to these findings, we have previously reported that a high expression level of IF1 in breast carcinomas is indicative of less chance to develop metastatic disease. This finding is especially relevant in the bad prognosis group of patients bearing triple-negative breast carcinomas. To investigate the molecular mechanisms that underlie the differential behavior of IF1 in breast cancer progression, we have developed the triple-negative BT549 breast cancer cell line that overexpresses IF1 stably. When compared to controls, IF1-cells partially shut down respiration and enhance aerobic glycolysis. Transcriptomic analysis suggested that migration and invasion were specifically inhibited in IF1-overexpressing breast cancer cells. Analysis of gene expression by qPCR and western blotting indicate that IF1 overexpression supports the maintenance of components of the extracellular matrix (ECM) and E-cadherin concurrently with the downregulation of components and signaling pathways involved in epithelial to mesenchymal transition. The overexpression of IF1 in breast cancer cells has no effect in the rates of cellular proliferation and in the cell death response to staurosporine and hydrogen peroxide. However, the overexpression of IF1 significantly diminishes the ability of the cells to grow in soft agar and to migrate and invade when compared to control cells. Overall, the results indicate that IF1 overexpression despite favoring a metabolic phenotype prone to cancer progression in the specific case of breast cancer cells also promotes the maintenance of the ECM impeding metastatic disease. These findings hence provide a mechanistic explanation to the better prognosis of breast cancer patients bearing tumors with high expression level of IF1.

## Introduction

Partial suppression of ATP production by mitochondrial oxidative phosphorylation and the concurrent metabolic reprogramming of the cell to an enhanced aerobic glycolysis is a hallmark feature of proliferating normal, cancer, and stem cells (1–6). This switch in metabolic phenotype warranties that a fraction of the glucose carbon skeletons available rather than being oxidize to CO_2_ could supply the anabolic precursors and reducing power that are needed to sustain proliferation (7–10). The ATP synthase is the enzyme that utilizes the proton electrochemical gradient generated by respiration for the synthesis of ATP (11, 12). Several mechanisms promote the rewiring of metabolism in human carcinomas at the level of the ATP synthase (13, 14). The main of the ones described are (i) the partial downregulation of the expression of the catalytic subunit of the ATP synthase (β-F1-ATPase) (7, 15, 16), which in solid carcinomas is exerted by the specific repression of β-F1-ATPase mRNA translation (14, 17, 18) and in chronic myeloid leukemia by hypermethylation and silencing of the promoter of the *ATP5B* gene (19) and (ii) by the inhibition of the activity of the ATP synthase mediated by the overexpression of the ATPase inhibitory factor 1 (IF1) (20–22).

The activity of IF1 as an inhibitor of the ATP synthase is regulated by matrix pH under conditions of mitochondrial de-energization (13, 23–25) and by the phosphorylation of S39 under several physiological situations such as progression through the cell cycle, hypoxia, rapid changes in metabolic demand, and cancer (26). In the specific case of breast carcinomas, we have recently described that IF1 is found essentially in its dephosphorylated form and hence able to bind and inhibit the ATP synthase activity of the enzyme (13, 26). Remarkably, the overexpression of IF1 in different cancer cells promotes the acquisition of a pro-oncogenic phenotype by inducing metabolic reprogramming to an enhanced glycolysis (21, 22, 27). Moreover, overexpression of IF1 triggers in mitochondria a concurrent reactive oxygen species (ROS) signal that switches on the NF-κB pathway favoring invasion and cell survival in colon and lung cancer cells (21, 27). Consistently, a high expression level of IF1 in human hepatocarcinomas (28) and in carcinomas of the lung (29), bladder (30), and stomach (31) and in gliomas (32) is a biomarker of bad prognosis for the patients. In sharp contrast to these findings, a high expression level of IF1 in breast carcinomas positively correlates with less chance to develop metastatic disease; in other words, it is a biomarker of good prognosis for breast cancer patients (21).

In this study, we have questioned the molecular bases that sustain why a high expression level of IF1 in breast carcinomas yields a biomarker of good prognosis for the patients despite favoring a phenotype prone to oncogenesis (21). The transcriptomic and phenotypic analysis of breast cancer cells expressing high levels of IF1 supports that a good prognosis in these patients is based on the poorer potential of the cells to migrate and invade, as a result of a better maintenance of the extracellular matrix (ECM) and epithelial phenotype.

## Materials and Methods

### Patient Specimens and IF1 Determinations

A collection of anonymized frozen tissue sections obtained from surgical specimens of 93 patients who had an operation for invasive breast carcinoma at the Hospital Universitario La Paz (HULP) between 1991 and 2000 was interrogated in previous studies for the expression of proteins of energy metabolism (33, 34). The expression of IF1 in these breast biopsies and the study of its correlation with patients’ survival have been partially described previously (21). The Cancer Survey Tissue Microarray (OriGene, Rockville, MD, USA) containing sections of formalin-fixed normal and tumor specimens of the breast were immunostained using the monoclonal anti-IF1 (1:200) antibody (22). Sections were counterstained with hematoxylin. Patient’s medical records were reviewed and identifiers coded to protect patient confidentiality.

### Generation of Cell Lines and Cell Cultures

The breast cancer BT549-luc cell line was kindly provided by Dr. Murakami from the Japanese Collection of Research Bioresources Cell Bank (#JCRB1373). The BT549-luc cell line is a triple-negative breast cancer cell that is estrogen receptor negative, progesterone receptor negative, and lacks epidermal growth factor receptor 2 (Her2/neu) overexpression. Cells were cultured in RPMI-1640 medium supplemented with 15% FBS and 1% penicillin/streptomycin in a 5% CO_2_ incubator. The pCDH-CMV-MCS-EF1-RFP + Puro cDNA Cloning and Expression Vector (SBI#CD616B-2) (SBI System Biosciences) was used to establish cells overexpressing the human ATPase IF1. The plasmid also expresses RFP for selection purposes. The human IF1 insert was obtained from the pCMV-Sport6-IF1 plasmid (22) by digestion with the *Not*I and *Eco*RI restriction sites and ligated with the linearized plasmid to generate the pCDH-CMV-MCS-EF1-RFP + PURO-IF1 plasmid. All the constructs were checked by sequencing. Stable cell lines derived by transfection with the pCDH-CMV-MCS-EF1-RFP + PURO empty vector (control, CRL) and the pCDH-CMV-MCS-EF1-RFP + PURO-IF1 were generated using lentivirus. For lentiviral transfection, viral particles were produced in HEK293T cells. They were cultured in DMEM 10% FBS. After the transfection of BT549 cells, stable transfectants were selected by adding 6 μg/ml puromycine (Invitrogen; Thermo Fisher Scientific, Inc.) to the growth medium.

### Cellular Lysis and Western Blotting

Cell lysis was performed with RLN-T buffer (RLN buffer plus 0.5% Triton X-100 and the complete protease inhibitors cocktail EDTA-free; Roche) at 20 × 10^6^ cells/ml for 5 min on ice. Extracts were centrifuged at 11,000 × *g* for 15 min at 4°C. The primary antibodies used were as follows: anti-IF1 (1:200) (22), e-cadherin (1:250, BD Biosciences), β1 integrin (1:2, kindly provided by Carlos Cabañas, CBMSO), β-catenin (1:500, BD Biosciences), vimentin (1:1,000, Cell Signaling), NF-κB p65 (1:1,000, Abcam), pIKBα (1:500, Cell Signaling), IKBα (1:1,000, Cell Signaling), α-tubulin (1:3,000, Sigma-Aldrich), β-F1-ATPase (1:25,000) and Hsp60 (1:2,000) from Ref. (35), SDH-B (1:500, Invitrogen),α-F1-ATPase (1:1,000, Molecular Probes), Core 2 of complex III (1:500, Abcam), MTCO2 (1:500, Abcam), VDAC (1:500, Abcam), and PYGM (1:1,000, Abcam).

### Subcellular Fractionation

Mitochondrial isolation was performed according to Acin-Perez et al. (36). In brief, cells were washed with PBS. Cellular pellets were homogenized in a glass–teflon homogenizer with seven volumes of hypotonic buffer (83 mM sucrose, 10 mM MOPS pH 7.2). After homogenization, the same volume of hypertonic buffer (250 mM sucrose, 30 mM MOPS pH 7.2) was added and nuclei and unbroken cells were removed by centrifugation at 1,000 × *g*. Mitochondria were obtained by centrifugation at 12,000 × *g* and washed in buffer A (320 mM sucrose, 1 mM EDTA, 10 mM Tris–HCl pH 7.4). Cytosolic fraction was collected in the supernatant. Mitochondria were further purified by centrifugation through a 0.8 M sucrose cushion in 10 mM Tris–HCl pH 7.5 0.1% BSA. Cellular, mitochondrial, and cytosolic proteins were loaded on SDS-PAGE, transferred onto nitrocellulose membranes, and immunoblotted against diverse antibodies.

### Determination of the H^+^-ATP Synthase Activity and Cellular ATP

The ATP synthetic activity was determined in digitonin-permeabilized cells following the detailed protocol in Ref. (37). Inhibition of the synthase activity of the H^+^-ATP synthase was accomplished by the addition of 30 μM oligomycin (OL). Cellular ATP concentrations were determined using the ATP Bioluminescence Assay Kit CLS II (Roche) following the manufacturer’s instructions.

### Glycolysis, Cellular Respiration, and ROS Production

The initial rates of lactate production were determined as an index of glycolysis by enzymatic determination of lactate concentrations in the culture medium (38). Oxygen consumption rates were determined in an XF24 Extracellular Flux Analyzer (Seahorse Bioscience) (22). Cells were seeded in the microplates, and incubated at 37°C and 5% CO_2_ for 24 h. The final concentration and order of injected substances was 6 μM OL, 0.75 mM DNP (2,4-dinitrophenol), 1 μM rotenone, and 1 μM antimycin. The intracellular production of hydrogen peroxide was monitored by flow cytometry using 5 μM 6-carboxy-2′,7′-dichlorodihydrofluorescein diacetate (DCFH2-DA) (Molecular Probes, Eugene, OR, USA) and incubated 30 min at 37°C (27). Cells were analyzed in a BD FACScan. For each analysis, 10,000 events were recorded.

### Gene Array Hybridization

Total RNA was extracted from BT549 cells using Trizol (Invitrogen, Carlsbad, CA, USA) followed by the Qiagen RNeasy (Qiagen, Valencia, CA, USA). Each RNA preparation was tested for degradation using the Agilent 2100 Bioanalyzer (Agilent Technologies, Palo Alto, CA, USA). Total RNA (200 ng) was amplified using One-Color Low Input Quick Amp Labeling Kit (Agilent Technologies) and purified with RNeasy Mini Kit (Qiagen). Preparation of probes and hybridization was performed as described in One-Color Microarray Based Gene Expression Analysis Manual Ver. 6.5, Agilent Technologies. Briefly, for each hybridization, 600 ng of Cy3 probes were mixed and added to 5 μl of 10× blocking agent, 1 μl of 25× fragmentation buffer, and nuclease-free water in a 25-μl reaction, incubated at 60°C for 30 min to fragment RNA, and stopped with 25 μl of 2× hybridization buffer. The samples were placed on ice and quickly loaded onto arrays, hybridized at 65°C for 17 h in a hybridization oven, and then washed in GE Wash Buffer 1 at room temperature (1 min) and in GE Wash Buffer 2 at 37°C (1 min). Arrays were dried by centrifugation at 2,000 rpm for 2 min. Slides were Sure Print G3 Agilent 8x60K Human (G4852A-028004). Images were captured with an Agilent Microarray Scanner and spots quantified using Feature Extraction Software (Agilent Technologies). Background correction and normalization of expression data were performed using LIMMA (39, 40). Linear model methods were used for determining differentially expressed genes. Each probe was tested for changes in expression over replicates by using an empirical Bayes moderated *t*-statistic (39). To control the false discovery rate *p*-values were corrected by using the method of Benjamini and Hochberg (41). The expected false discovery rate was controlled to be <5%. Hybridizations and statistical analysis were performed by the Genomics Facility at Centro Nacional de Biotecnologia (Madrid, Spain).

### Gene Data Analysis

A gene list was made by selecting genes with a fold change ≥1.5 or ≤−1.5 between control and IF1-overexpressing cells. A corrected *p*-value ≤0.05 was considered statistically significant. GeneCodis3 tool was used to perform gene set enrichment analysis with GeneCodis (42) to infer the main biological functions associated with IF1 overexpression using KEGG and Panther pathways enrichments. Gene set enrichment by Ingenuity Pathway Analysis (Qiagen) was also used to determine the specific canonical signaling pathways and related diseases and functions affected. A Fisher’s exact right-tailed test identified significantly enriched pathways, and a *z* score was computed to determine whether the pathway was activated or inhibited at each stage.

### Quantitative Reverse Transcription Polymerase Chain Reaction (PCR) Analysis

Reverse transcription reactions were performed using 1 μg of total RNA and the High Capacity Reverse Transcription Kit (Applied Biosystems; Thermo Fisher Scientific, Inc.). Real-time PCR was performed with an ABI PRISM 7900HT SDS (Applied Biosystem) and Power SYBR Green PCR Master Mix (Applied Biosystems). The following primers were used: ACTA2 (NM_001141945), DCN (NM_001920), ITGB1 (NM_002211), POSTN (NM_001135934), TGFB3 (NM_003239), SERPINB7 (NM_001040147), SERPINB11 (NM_080475), VCAN (NM_001126336), and WNT2B (NM_004185). All primers provided by Sigma-Aldrich.

### Cellular Proliferation and Cell Death Assays

Cellular proliferation was determined by counting the number of cells/well after 24, 48, and 72 h of culture. For cell death assays, 30,000 cells/well were seeded and treated with 1 μM staurosporine (STS) or 120 μM hydrogen peroxide during 24 h. Cell death was determined by flow cytometry after staining with annexin V (ApoScreen FITC; Southern #10010-02) (27).

### Soft Agar, Wound Healing, and Invasion Assays

For soft agar assay, 2,000 cells were added to 750 μl of complete growth media with 0.5% agarose and layered onto a 750 μl bed of complete growth media plus 0.7% of agar on a 12-well plate. After 5 weeks, viable colony numbers were stained with 0.5% crystal violet in 4% paraformaldehyde and counted using ImageJ software. For wound healing assays, confluent cell monolayers were mechanically disrupted with a sterile pipette tip to produce a clean uniform scratch. The wells were photographed every 30 min. Gap distances were analyzed with ImageJ software. Corning BioCoat Matrigel Invasion Chambers were used to quantify the cellular invasive capacity. A total of 2 × 10^4^ cells were seeded in 1% FBS and chemoattraction perform during 72 h in 20% FBS.

### Statistical Analysis

The results shown are the means ± SEM of four to six biological replicates. Statistical analysis was performed by Student’s *t*-test, Mann–Whitney *U* test, and/or Kruskal–Wallis as appropriate. Tests were calculated using the SPSS 13.0 software package (IBM, Chicago, IL, USA). Survival curves were derived from Kaplan–Meier estimates and compared by log-rank test. Statistical tests were two sided at the 5% level of significance.

## Results

### Overexpression of IF1 in Human Breast Carcinomas

**Figure 1:**
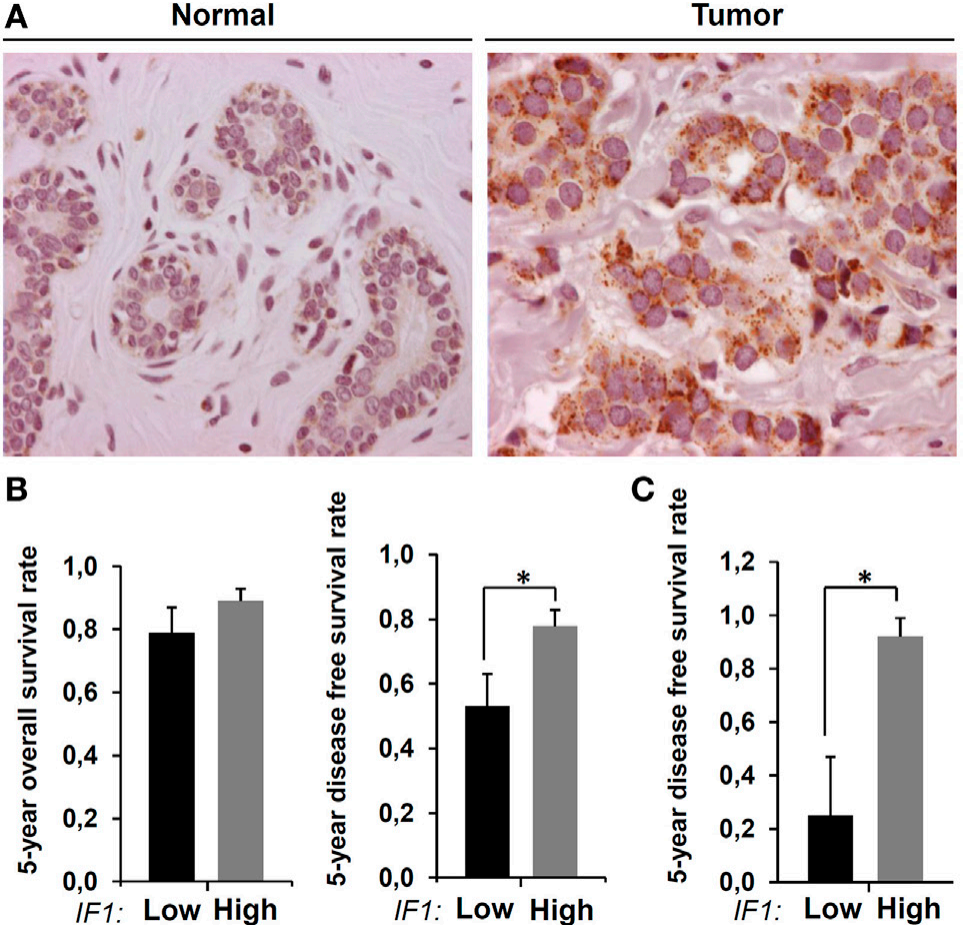
**Expression of inhibitory factor 1 (IF1) in human breast carcinomas**. **(A)** Representative immunohistochemistry of IF1 expression in normal and tumor tissue of the breast. **(B)** The histograms show the Kaplan–Meier 5-year overall and disease-free survival rates (mean ± SEM) for the cohort of 93 breast cancer patients stratified by the tumor expression level of IF1 (21). **(C)** Same as above for the 18 hormonal receptor negative subgroup of carcinomas contained in the cohort. Protein samples from normal and tumor breast samples were analyzed by western blot for the expression level of IF1 as indicated previously (21). *p = 0.03 and *p = 0.001 show the log-rank test *p*-value in **(B)** and **(C)**, respectively.

We have previously documented in a large cohort of breast cancer patients (33) that IF1 is highly overexpressed in breast carcinomas (21) [for details, see supplemental Table 1 in Ref. (21)]. These results have been further confirmed in another large cohort of breast carcinomas (43). Figure 1A provides an illustrative example of the changes taking place in IF1 expression in breast by oncogenesis. The normal breast tissue shows negligible expression of IF1, whereas a very high expression level of the protein is observed in the carcinomas. Figure 1B shows that the tumor expression level of IF1 has no relevant influence on the 5-year overall survival rate in breast cancer patients. However, when the content of IF1 in the carcinoma is high (21), it significantly associates with an increase in disease-free survival of the patients (Figure 1B). The association of high IF1 levels with a lesser chance to develop metastatic disease is magnified in the poor prognosis group of triple-negative breast carcinomas (Figure 1C) (21). Overall, these findings suggest that high IF1 levels in breast carcinomas in some way correlate with a decrease of metastasis despite supporting a pro-oncogenic metabolic phenotype (21, 22, 27).

### Energy Metabolism of IF1-Overexpressing BT549 Cancer Cells

**Figure 2:**
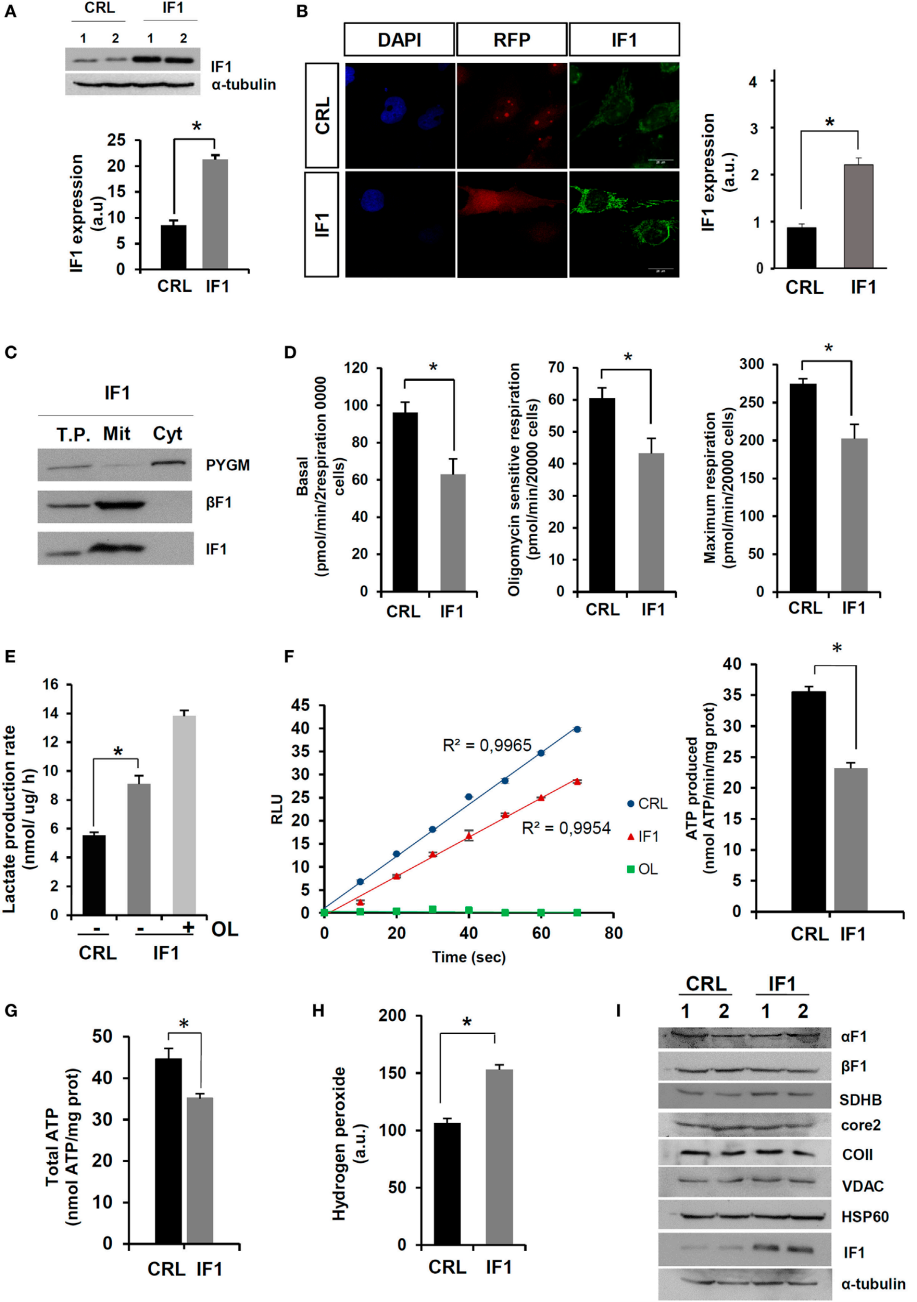
**Energy metabolism of inhibitory factor 1 (IF1)-overexpressing BT549-luc cells**. **(A,B)** The histograms show the quantification of IF1 expression in two different biological replicates (1 and 2) by immunoblotting **(A)** and immunofluorescence microscopy **(B)**. Nuclei: blue, DAPI staining; transfection marker: red fluorescent protein, RFP; mitochondria: green fluorescence, IF1. **(C)** Overexpressed IF1 is exclusively localized in mitochondria (Mit). T.P., total cellular protein; Cyt, cytosolic protein; PYGM, glycogen phosphorylase; βF1, β-F1-ATPase. **(D)** Rates of basal, oligomycin (OL) sensitive, and maximum respiration determined in the X24 Seahorse Flux Analyzer. **(E)** Initial rates of lactate production in the absence (−) or presence (+) of 6 μM OL. **(F)** Plot, kinetic representation of the linear production of ATP in relative light units (RLU) in CRL (blue) and IF1 (red) expressing digitonin-permeabilized cells. The inhibition of ATP synthase activity by the addition of 30 μM OL is shown in red. Histograms shows the ATP synthetic activity. **(G)** Cellular ATP concentration in CRL and IF1-overexpressing cells. **(H)** Cellular hydrogen peroxide detection with DCFH2-DA. CRL, control; IF1, IF1-overexpressing cells; a.u., arbitrary units. **(I)** Representative western blots of the expression of mitochondrial proteins (α-F1-ATPase, β-F1-ATPase, SDH-B, Core 2, COII, VDAC, Hsp60, and IF1) and tubulin in two different replicates of CRL and IF1-overexpressing cells. The results shown are means ± SEM of four to six different replicates. *p ≤ 0.05 when compared to CRL by Student’s *t*-test.

In order to investigate the molecular basis of this paradox, we developed stable IF1-overexpressing breast cancer cells (IF1-cells). For this purpose, we used the triple-negative breast cancer cell line BT549-luc. After transfection and selection, the overexpression of IF1 was confirmed both by immunoblotting (Figure 2A) and immunofluorescence microscopy (Figure 2B). The overexpressed transgene was localized in mitochondria (Figures 2B,C). Consistent with previous findings in transient expression experiments (21), the lentivirus-driven overexpression of IF1 significantly diminished the rates of cellular respiration (Figure 2D). Moreover, and when compared to controls, the rates of glycolysis were significantly augmented in IF1-cells (Figure 2E), further supporting that IF1 is interfering with the mitochondrial production of ATP by the ATP synthase (21, 22, 27). In fact, the expression of IF1 partially inhibited the ATP synthase activity as determined in permeabilized cells (Figure 2F) resulting in a diminished cellular content of ATP (Figure 2G). However, it should be noted that the rates of aerobic glycolysis in IF1-cells can still be overstimulated by incubation with OL (Figure 2E), indicating that the overexpression of IF1 is not enough to inhibit all the ATP synthase present in the cell. In agreement with previous reports (21, 27), the IF1-mediated inhibition of the ATP synthase resulted in a significant increase in basal cellular ROS levels (Figure 2H) without affecting the expression of other mitochondrial proteins (Figure 2I). Altogether, these results confirmed the biological activity of IF1 as a regulator of cellular energy metabolism in agreement with previous results.

### IF1-Mediated Gene Expression

**Figure 3:**
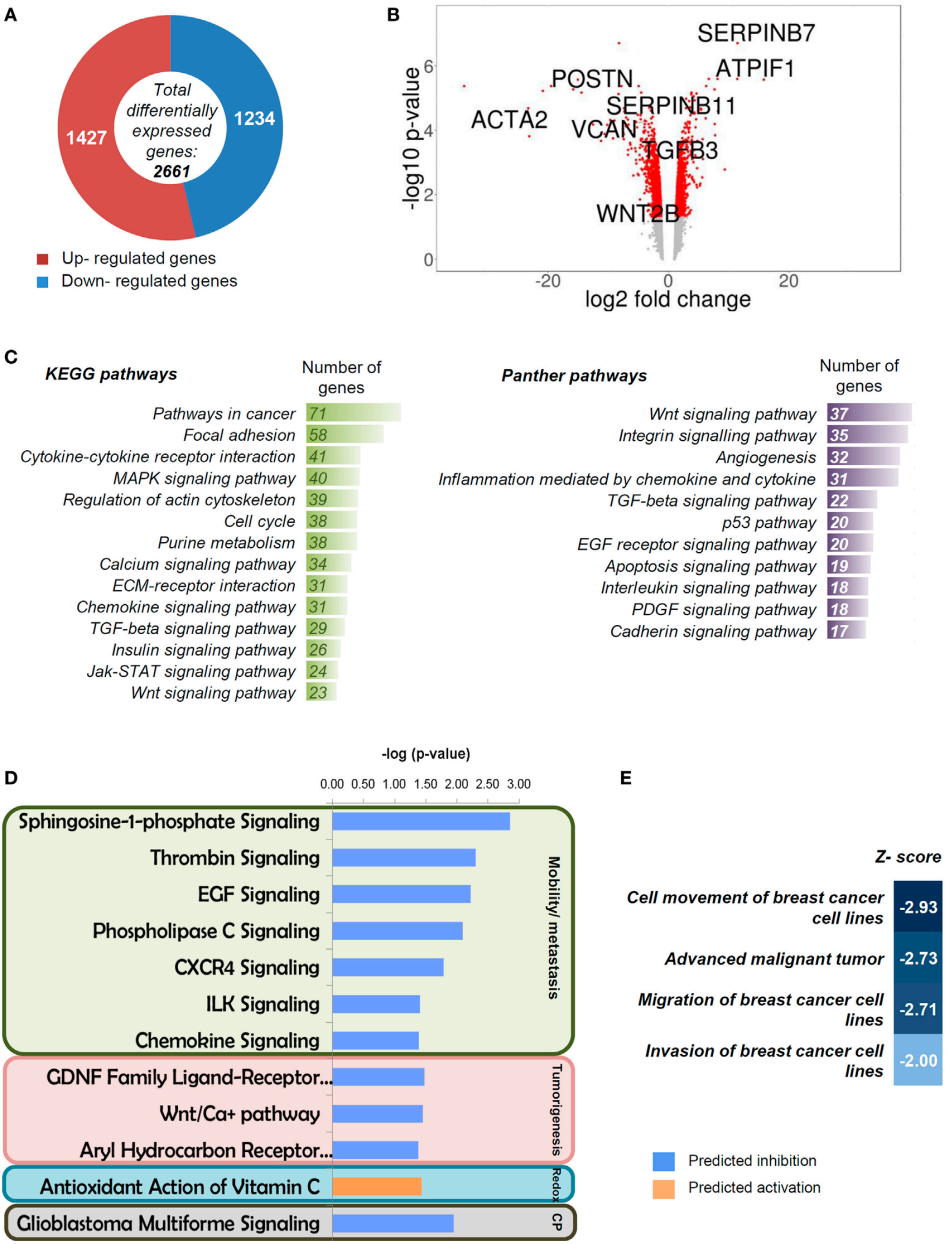
**Transcriptome of breast cancer inhibitory factor 1 (IF1)-overexpressing cells**. **(A)** Representation of the total number of significantly affected genes by IF1 overexpression when compared to controls using Agilent 8x60K Human arrays. **(B)** Volcano plot with some relevant genes indicated. *X* axis represents the expression fold change of the affected genes, and the *Y* axis represents −log10 of the FDR values. **(C)** Gene enrichment analysis, showing the information related to KEGG and Panther databases. **(D,E)** Pathways **(D)** and diseases and functions **(E)** affected by IF1 overexpression as revealed by the IPA ingenuity tool. *Z*-score indicates the overall predicted activation/inhibition state of the function. CP, other cancer processes (glioblastoma multiforme signaling).

Analysis of the transcriptome of control (empty vector) and IF1-overexpressing breast cancer cells rendered 2,661 genes differentially expressed (*p* ≤ 0.05; fold change ≥1.5; LIMMA analysis) (Figure 3A; Table S1 in Supplementary Material). From the global list of significant genes (Table S1 in Supplementary Material), 1,427 were upregulated and 1,234 were downregulated (Figure 3A). A volcano plot that combines the statistical significance with the magnitude of change illustrates the distribution of the IF1-regulated genes further emphasizing some of the genes that display large magnitude of significant change (Figure 3B). Consistently, the *ATPIF1* gene was found significantly overexpressed in the transcriptome of IF1-cells (Figure 3B). A pathway enrichment analysis using the GeneCodis tool (42) against KEGG and Panther pathways databases highlighted that most of the affected pathways on IF1-cells were related to cancer (Figure 3C). In the case of KEGG database, the affected pathways revealed association with the ECM (focal adhesion, ECM–receptor interaction, and regulation of actin cytoskeleton), intercellular communication (cytokine–cytokine receptor interaction and MAPK signaling, calcium signaling, and chemokine signaling pathways), and cell cycle (Figure 3C). In the case of Panther database, similar groups appeared with the addition of angiogenesis and apoptosis (Figure 3C). Moreover, we performed an additional enrichment analysis with IPA ingenuity tool in order to complement the information with a prediction of the activation/repression status of the affected pathways. The results obtained pointed out that the majority of the canonical pathways affected in IF1-cells were related to cancer (Figure 3D) and, more specifically, to cellular mobility/metastasis and tumorigenesis in agreement with the previous enrichment analysis. Remarkably, these functions were predicted to be inhibited in IF1-cells when compared to controls (Figure 3D). Moreover, the IPA analysis of diseases and functions highlighted that migration and invasion of breast cancer cells were specifically inhibited in IF1-overexpressing cells (Figure 3E). Overall, these results suggest that IF1 overexpression could favor the generation of a cellular phenotype with less mobility and invasiveness, which could underpin the lower metastasis and better prognosis of breast cancer patients bearing tumors with high expression level of the protein.

**Figure 4:**
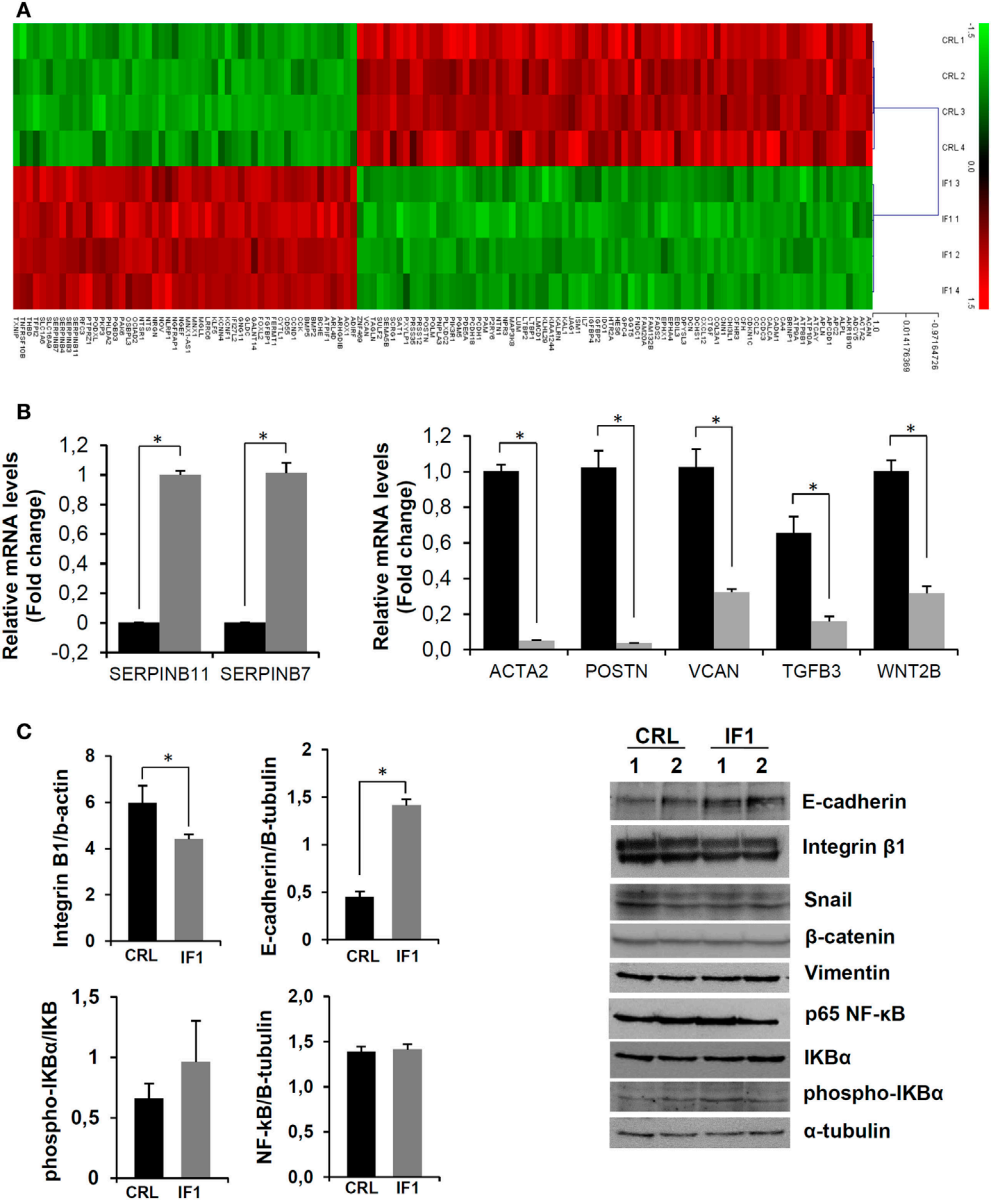
**Breast cancer inhibitory factor 1 (IF1)-overexpressing cells have a less migratory and invasive phenotype**. **(A)** The heatmap shows the transcriptome of the 138 differentially expressed genes between control (CRL) and IF1-overexpressing cells that meet the Bonferroni correction. Four different samples of each cell type (CRL and IF1) were included in the Agilent 8x60K Human arrays. **(B)** Quantitative reverse transcription PCR validation of up and downregulated genes in the microarray analysis in CRL (closed bars) and IF1-overexpressing (gray bars) cells. **(C)** Protein expression of extracellular matrix and epithelial–mesenchymal transition-related factors detected by immunoblotting. **(B,C)** The histograms show the quantification of mRNA **(B)** and protein **(C)** expression as the means ± SEM. *p ≤ 0.05 when compared to CRL by Student’s *t*-test.

Tables S2 and S3 in Supplementary Material list the 52 upregulated and 79 downregulated genes in IF1-cells when compared to controls that meet the more restrictive multiple correction test of Bonferroni (Figure 4A). Pathway enrichment analysis using the GeneCodis tool with the set of 138 genes also confirmed that cellular movement and cell-to-cell signaling and interaction are affected in IF1-overexpressing breast cancer cells.

### IF1 Overexpression Affects the Expression of ECM-Related Players

The results of the transcriptomic analysis were validated by the quantification of the expression of some of the genes involved in ECM and its signaling by quantitative PCR (Figure 4B). Consistently, *SERPINB7* and *SERPINB11*, which are members of the superfamily of inhibitors of extracellular proteases, were highly up regulated in IF1-cells (Figure 4B). On the other hand, a significant diminished expression of *ACTA2*, encoding a member of the actin protein family important for cell movement and contraction; *POSTN* which encodes periostin—a component of the ECM involved in the organization of collagens; and *VCAN*—a member of the versican proteoglycan family which is involved in cell adhesion, were found downregulated in IF1-overexpressing cells (Figure 4B). Likewise, the expression of signaling molecules such as the cytokine TGFB3, which is involved in the regulation of cell adhesion, of the ECM, and of *WNT2B* (formerly *WNT13*), encoding a glycoprotein of the wingless secreted signaling factors that promote epithelial–mesenchymal transition (EMT), was significantly downregulated in IF1-overexpressing cells (Figure 4B). Overall, these results support that a low expression of IF1 in breast cancer cells favors remodeling of the ECM to facilitate cellular migration and metastasis.

Next, we studied by immunoblotting the expression of some proteins commonly associated with the modification of the ECM, the induction of EMT, and cell migration. The results revealed no differences in vimentin or β-catenin levels between control and IF1-overexpressing cells (Figure 4C). Likewise, the NF-κB and Snail pathways—two pathways frequently activated in cancer—were not significantly affected by the overexpression of IF1 in breast cancer cells (Figure 4C). However, the levels of E-cadherin, an adhesion molecule involved in the maintenance of cellular and epithelial tissue integrity, was significantly upregulated in IF1-overexpressing cells (Figure 4C). Furthermore, integrin-β1—an adhesion molecule related to migration, extravasation, and metastasis (44)—levels were found significantly upregulated in control when compared to IF1-overexpressing cells (Figure 4C). Overall, the results reveal that the overexpression of IF1 in breast cancer cells supports the maintenance of ECM and tissue integrity.

### IF1-Overexpressing BT549 Cells Have Less Tumorigenic and Migration Capacity

**Figure 5:**
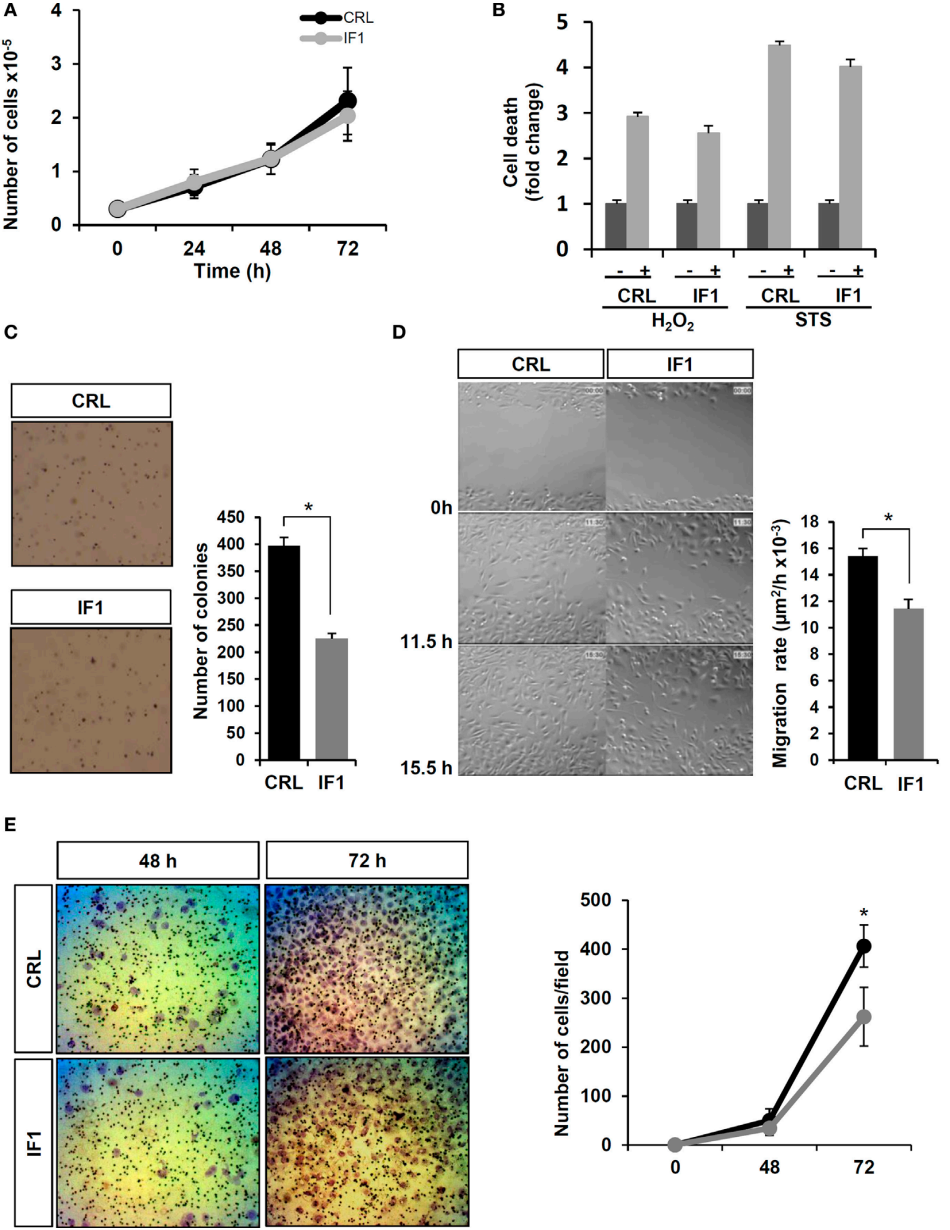
**Breast cancer inhibitory factor 1 (IF1)-overexpressing (gray line and bars) cells are more vulnerable and migrate and invade less than control (CRL, black and closed bars) breast cancer cells**. **(A)** Cellular proliferation at 24, 48, and 72 h. **(B)** Cell death after 24 h of priming the cells with 1 μM staurosporine (STS) or 120 μM hydrogen peroxide. *p < 0.01 when compared to non-treated by Student’s *t*-test. **(C)** Representative images of the anchorage-independent growth in soft agar. **(D)** Representative time frames of the wound healing video assays. **(E)** Representative images of the matrigel invasion assays at 48 and 72 h. The histograms **(C,D)** and graph **(E)** show the quantification as the means ± SEM; *p ≤ 0.05 when compared to CRL by Student’s *t*-test.

Assessment of the rates of cellular proliferation (Figure 5A) and of cell death after hydrogen peroxide or STS treatment (Figure 5B) revealed no relevant differences between control and IF1-expressing cells. Interestingly, soft agar colony-formation assays showed that IF1-cells had a significant less capacity to grow and form colonies in the anchorage-independent assay (Figure 5C), suggesting a lower tumorigenic potential. To verify the migration ability of the cells, wound healing assays were carried out (Figure 5D). The results revealed that control cells started filling and fully occupied the scratched area earlier than IF1-cells (Figure 5D; see Video S1 in Supplementary Material), indicating that IF1-overexpressing cells had less migration ability than control cells. Similarly, matrigel invasion assays also revealed that control cells had a higher invasive capacity than IF1-cells (Figure 5E). Overall, these results suggest that IF1 overexpression in breast cancer cells induces a less aggressive phenotype by diminishing the migration and invasive capacities of the cells. This finding agrees with the fact that breast cancer patients with elevated tumor levels of IF1 had less metastatic disease.

## Discussion

Triple-negative breast cancers are defined as tumors that lack expression of estrogen receptor, progesterone receptor, and *HER2* (45). Triple-negative carcinomas represent 15–20% of tumors in women with breast cancer, have a relatively poor outcome, and are refractory to hormone and epidermal growth factor receptor type 2 (*HER2*) therapies (45). Herein, we report that the overexpression of IF1 in human breast carcinomas, especially in the subgroup of triple-negative breast carcinomas predicts a lower risk for metastatic disease (21). This finding is counterintuitive since (i) the overexpression of IF1 inhibits mitochondrial respiration and enhances glycolysis, which is a metabolic phenotype that is enforced in proliferating invasive cells (2, 3, 5, 6), (ii) a high expression level of IF1 has been recently reported as biomarker of bad prognosis in human hepatocarcinomas (28) and in carcinomas of the lung (29), bladder (30), and stomach (31) and in gliomas (32), and (iii) the overexpression of IF1 in the liver of transgenic mice significantly contributes to an increase in hepatocarcinogenesis (46). To investigate the molecular mechanisms that might support the “non-canonical” behavior of IF1 in breast cancer, which might open up potential new trends in diagnosis and treatment of breast cancer patients, we developed the triple-negative breast cancer BT549-luc cell line that stably overexpresses IF1. Consistent with previous results in transient transfection experiments in breast cancer cells (21), we show that stably overexpressing IF1-cells partially suppressed respiration, induced aerobic glycolysis, and showed and enhanced basal ROS levels when compared to control cells. Transcriptomic analysis suggested that IF1-cells have a less aggressive phenotype when compared to controls because they concertedly upregulate the expression of genes involved in repression of ECM dismantling and overexpress genes that support ECM. These findings have been validated at both the transcriptome and proteome levels and by *in vitro* cellular assays that illustrate that IF1-cells show a diminished ability for anchorage-independent growth and migratory and invasive capacities.

The overexpression of IF1 in prevalent human carcinomas including breast cancer has been shown to be unrelated to major changes in mRNA availability (21). Thus, the accumulation of IF1 in breast carcinomas, which is a protein with very short half-life (21), should result from alterations in the synthesis and/or degradation rates of the protein brought about by oncogenesis (21). Posttranslational modifications of IF1 could also contribute to the differential regulation of the turnover of the protein in cancer. In this regard, IF1 is a mitochondrial protein that experiences several posttranslational modifications such as phosphorylation, acetylation, glycosylation, and succinylation [for review, see Ref. (13)]. In the case of protein phosphorylation it renders a protein that is inactive as an inhibitor of the ATP synthase, because phosphorylation prevents IF1 binding to the enzyme (26) and, in most of the breast, colon, and lung carcinomas, analyzed IF1 is present in its dephosphorylated active form (26). Interestingly, the glycosylation of IF1 has also been described in breast carcinomas (43). Hence, we suggest that the study of IF1 turnover is a most relevant aspect of the pathophysiology of IF1 deserving a thorough future investigation in breast cancer.

In gastric (31) and bladder (30) cancer, an increased expression of IF1 has been shown to promote cellular proliferation. Likewise, the overexpression of IF1 in colon cancer cells triggers the transcriptional activation of the NF-κB pathway, thereby favoring proliferation and preventing cell death (27). Similarly, less apoptosis and an enhanced proliferation have been observed in induced hepatocarcinomas in transgenic mice overexpressing a constitutively active mutant of IF1 (46). Prevention of cell death by the overexpression of IF1 has also been observed in lung (21) and gastric (31) cancer cells. However, we show that the overexpression of IF1 in breast cancer cells has no significant impact in cellular growth and cell death response to STS and hydrogen peroxide. Interestingly, the overexpression of IF1 in hepatocarcinomas (28) and in gliomas (32) also promotes the activation of the NF-κB pathway that, by triggering the activation of Snail favors EMT and hence cellular migration and invasion. However, breast cancer IF1-cells did not show any relevant activation of the NF-κB and Snail pathways. Moreover, no relevant changes were observed in the expression of β-catenin and vimentin, respectively, representing epithelial and mesenchymal markers. In fact, breast cancer IF1-overexpressing cells provided a phenotype completely opposite to that summarized above for other cancer cells. The phenotype of IF1-cells is compatible with the inhibition of cell migration and the maintenance of the ECM. Consistently, members of the Serpin B clade are overexpressed in IF1-cells being some of its members directly related with suppression of migration and invasion in breast cancer (47), whereas its downregulation is associated with the aggressiveness of squamous cell carcinomas (48, 49). On the other hand, (i) *ACTA2* which has been related to increased cell motility in breast cancer and other cancer types (50, 51), (ii) *POSTN*, a protein secreted by cancer cells that has been described to facilitate cell motility and was recently purposed as an interesting target for prevention and treatment of breast tumor metastasis (52, 53), and (iii) *VCAN*, known to enhance tumorigenesis and cell mobility, invasion, and survival of breast tumors (54), are all dramatically silenced in IF1-overexpressing breast cancer cells. Besides, we found that IF1 overexpression increased the presence of the epithelial marker E-cadherin involved in maintenance of cell and epithelial tissue integrity and inversely correlated with invasion and metastasis (55, 56). Furthermore, integrin β1 an adhesion molecule (55) related to migration, extravasation, and metastasis (44) is found significantly reduced in IF1-cells. Therefore, IF1 overexpression in breast cancer cells seems to promote the maintenance of ECM and tissue integrity diminishing their tumorigenic potential in agreement with the observed less migrating and invasive phenotype and consistent with the observation that IF1 overexpression is a biomarker of better prognosis in breast cancer patients (21). Moreover, our findings are also consistent with the idea that metastatic breast cancer cells are those with low IF1 expression level, in agreement with the observation that IF1 expression is significantly reduced in lymph node metastasis when compared to the primary tumors (43).

Growth in soft agar, wound healing, and matrigel invasion assays are *in vitro* approaches to assess the transformation potential of the cells by determining its anchorage-independent ability to grow or the cellular motility and invasiveness, which are related to the metastatic ability of neoplastic cells. Unfortunately, the BT549-luc cell line did neither develop orthotopic tumors nor gave rise to metastatic disease after tail vein injection when the cells were implanted into nude mice to assess tumor growth and metastatic potential *in vivo*. Therefore, the underlying IF1-mediated regulatory mechanism promoting tissue integrity in breast cancer *in vivo* remains to be elucidated. However, we suggest that TGF-β and Wnt signaling pathways whose activation is complex and known to promote EMT in different carcinomas including ovarian and breast cancer (57, 58), and highlighted in this study as downregulated in the transcriptomic analysis, might play a role in this regard. Overall, these findings indicate that IF1 signaling is cell type specific and suggest that any potential cancer therapy using IF1 as a target should be tailored having this point into consideration.

In conclusion, our study confirms IF1 as a regulator of cellular energy metabolism that favors an enhanced glycolysis in breast cancer cells but, at variance with the findings reported for IF1 in other carcinomas, it promotes the maintenance of ECM avoiding metastatic disease. These results further provide a mechanistic explanation to the observed positive correlation existing between high IF1 levels in breast carcinomas and the lower metastasis observed in these patients. Overall, the findings strongly support a stringent tissue specific function for IF1 in cancer that should be taken into consideration when considering the development of future therapies using energy metabolism as a target for cancer treatment.

## Ethics Statement

All procedures were conducted in accordance with the recommendations of the Comités Éticos de Investigación Clínica del Hospital Universitario La Paz (HULP) and the Universidad Autónoma de Madrid, with written informed consent from all subjects. All subjects gave written informed consent in accordance with the Declaration of Helsinki. The project was approved by the Ethical Committees of both the Review Boards of HULP (PI-352) and the Universidad Autónoma de Madrid (CEI-24-571).

## Author Contributions

LG-L, CN-T, CC-M, IM-R, BS, and LG-Llo performed research and analyzed data; LG-L and JC designed research, analyzed data, and wrote the paper. All the authors read, contributed, and approved the final manuscript.

## Conflict of Interest Statement

The authors declare that the research was conducted in the absence of any commercial or financial relationships that could be construed as a potential conflict of interest. The reviewer, AR, and handling editor declared their shared affiliation, and the handling editor states that the process nevertheless met the standards of a fair and objective review.

## Supplementary Material

Table S1 **Raw transcriptomic data of IF1 overexpressing cells compared to control cells**.

Video S1 **Representative wound healing video assay of control (to the left) and IF1 overexpressing (to the right) cells**.
